# Nutrivigilance: the road less traveled

**DOI:** 10.3389/fphar.2023.1274810

**Published:** 2023-10-11

**Authors:** Vijay Ronit Luthra, Hale Z. Toklu

**Affiliations:** Burnett School of Biomedical Sciences, College of Medicine, University of Central Florida, Orlando, FL, United States

**Keywords:** nutrivigilance, nutravigilance, nutraceuticals, nutrition support, adverse reactions, pharmacovigilance, adverse event report, phytovigilance

## 1 Introduction

### 1.1 Nutrivigilance/nutravigilance

The term “pharmacovigilance” defines the activities related to the collection, detection, assessment, monitoring, and prevention of adverse reactions (ADR) due to pharmaceuticals. An ADR is any response to a drug which is noxious and unintended, including lack of efficacy (Toklu and Mensah, 2016). The word “pharmacovigilance” is derived from pharmakon (drug in Greek) and vigilare (keep an eye on/monitor in Latin). Recently, the spectrum of this sort of “-vigilance” broadened to include safety of herbal products, cosmetics, and nutraceuticals (Chauhan et al., 2013; Schmitz et al., 2013; Toklu, 2016; Toklu et al., 2019). Furthermore, the prefixes nutra- and nutri-seem to interchangeably refer to the same idea, with prefix choice being primarily a regional spelling issue based on common language vowel structure. In a 2014 paper, Schmitz, et al. defined nutravigilance specifically as “the science and activities relating to the detection, assessment, understanding and prevention of adverse effects related to the use of a food, dietary supplement, or medical food” (Schmitz et al., 2013). Nutrivigilance is defined as “a set of activities and actions related to the detection, definition, and assessment of side effects that occur when consuming food and nutritional supplements” (Malve and Bhalerao, 2023). Indeed, many papers choose one spelling or the other, but with no differential in granular detail of the particular vigilance involved. Practically, both versions of the word point to the same idea; for the sake of consistency, we have used nutrivigilance in this paper.

Nutrivigilance is a term used to describe the monitoring of adverse effects related to the use of dietary supplements, functional foods, and other nutraceuticals. It involves the systematic collection, analysis, and evaluation of information on adverse effects associated with the use of these products. Nutrivigilance plays a critical role in ensuring safety and efficacy and is a vital component of any comprehensive public health strategy. In the absence of regulations regarding ostensibly nutritional products, however, consumers are forced to rely on a sponsoring company’s evaluation and presentation of their product, or outside groups acting in watchdog roles, in order to make informed decisions about which products are safe and, perhaps more importantly, even useful (Malve and Bhalerao, 2023).

In recent years, with the explosion of more and more products that claim to enhance health in some manner, there has been a growing interest in nutrivigilance in both the United States (US) and Europe (Nasri et al., 2014; Lüde et al., 2016; Morgovan et al., 2019). This paper will examine the state of nutrivigilance in these two regions and discuss the need, challenges, and opportunities for improving nutrivigilance in the future. As regulation plays an important role within and between the US and Europe, the paper also examines complications that can occur in the field of nutraceuticals, which may add a layer of complexity and often difficulty in adopting and amplifying tougher nutrivigilance stances and their impact.

The primary argument for this nutrivigilance is that any substance taken internally or applied cosmetically to a body’s exterior, whether serving an explicitly medical purpose or not, should be evaluated both for efficacy and for safety before reaching the hands of a consumer. The problem is that US law does not have provisions requiring the approval of these products prior to commercialization.

### 1.2 Nutraceuticals

What classifies as a nutraceutical? Intended as a blend of the terms nutritional and pharmaceutical, “nutraceutical” gathers together substances that are valued not only for their nutritive contribution, such as calories, vitamins, or minerals, but certain extra health benefits–whether they are real or merely claimed (Nasri et al., 2014). Coined in 1989 by the Foundation for Innovation in Medicine, nutraceuticals are non-specific biological therapies that are intended to foster general wellbeing, control chronic symptoms, or prevent later uprisings of disease or adverse circumstances in the long term (Malve and Bhalerao, 2023). This focus on the prevention of eventual problems is of main importance to adherents of the nutraceutical field.

### 1.3 Classification of nutraceuticals

The categories that nutraceuticals are sorted into generally depend on the source of their provenance, which are essentially natural, pharmacological conditions, and/or chemical constitution of the product (Nasri et al., 2014; Malve and Bhalerao, 2023). To better understand the need for nutrivigilance, it is important to illustrate the general categories of nutraceuticals, which reveal the wide net that a system of evaluation needs to cast in order to be effective: dietary supplements, nutrients, herbal supplements, animal-based supplements, and functional foods.

While these categories provide useful umbrellas for common provenance, many supplements fit into more than one category. Flaxseed oil provides essential Omega-3 fatty acids (nutrients), but also falls into plant-based herbal supplements. Thus, the classification outline provides only a broad understanding of supplement types, while individual supplements may fall under multiple categories based on their provenance or chemical structure.

Dietary supplements are regulated by the Food and Drug Administration (FDA) as food products, but the rules are not the same as with drugs and other food items. These supplements contain specific nutrients that are derived from other food products and are typically contained in a liquid, capsule, powder or pill form. Examples include prebiotic and probiotics, certain useful enzymes, and fiber supplements.

Standard nutrients are the essential nourishing elements of sustaining healthy life, which users may take in support of their regular diets. These can include vitamins, omega fatty acids in fish or flaxseed oil, minerals such as zinc or potassium, collagen peptides and amino acids.

Herbal supplements are derived from plants and their oils, roots, seeds, berries, or flowers. Used for many centuries, herbals are thought to have unique healing properties. Examples here are green tea capsules, chamomile tea, echinacea, and ginkgo. Antioxidants such as resveratrol are also extracted from plants.

Animal-based supplements are any supplement derived specifically from animal products or tissue which might be useful to humans. Examples here include collagen peptides, shark cartilage, glucosamine and chondroitin, apitherapy products such as honey or royal jelly, and the digestive enzymes lipase and pepsin, often sourced from lambs and calves.

Functional foods refer specifically to whole foods on their own, or that have been fortified with specific vitamins or minerals, in addition to other components of a human diet which are thought to reduce certain risks in terms of chronic disease. These foods also are purported to hold unique health benefits beyond what the food would typically suggest in terms of its nutrients. Examples of functional foods include apple cider vinegar, protein powders, mushroom extract, or seaweed moss.

**FIGURE 1:**
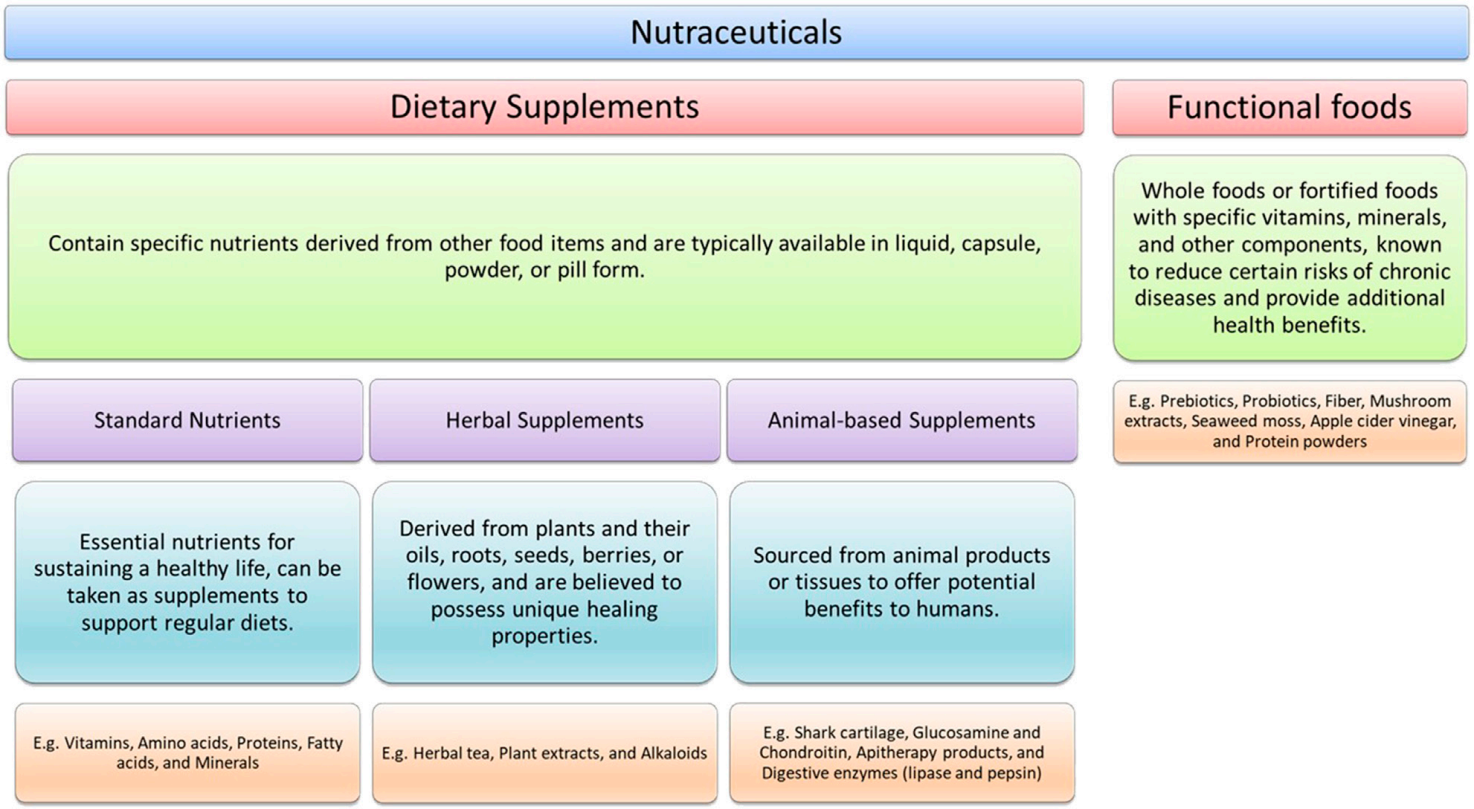
Classification of nutraceuticals.

There are various ways to categorize these substances based on function or composition. We have chosen to present these substances as nutraceuticals as shown in Figure 1.

### 1.4 What is the current regulatory scheme in the US?

The FDA is responsible for enforcing the laws and regulations governing the production, marketing, and use of dietary supplements. In general, FDA is limited to post market enforcement. Currently, the FDA does not have the legal authority to approve dietary supplements for safety and effectiveness. Additionally, the FDA does not evaluate the claims made by companies about the dietary supplements that they manufacture prior to the introduction of these supplements into the marketplace. In fact, manufacturers can market many dietary supplements without first notifying the FDA (FDA, 2022a; FDA, 2022d; FDA, 2023).

Manufacturers of dietary supplements must ensure their products are safe before marketing them to consumers and also comply with labeling and quality assurance requirements. The FDA inspects facilities for compliance and monitors adverse event reports. When public health concerns arise about the safety of a dietary supplement, the FDA has the authority to take action to protect the public.

To facilitate the reporting of safety concerns to the FDA by the general public, the agency created an online reporting platform on the FDA website called the Safety Reporting Portal (SRP) (FDA, 2022d). Federal law only requires manufacturers of dietary supplements to report serious adverse events to the FDA (FDA, 2023). The FDA, therefore, likely does not receive reports of all adverse events that come with supplement use and operates on limited knowledge about the efficacy and safety of these purportedly beneficial supplements.

In recent years, however, the FDA has taken steps to improve nutrivigilance. For example, in 2022 the FDA issued a document on how to conduct “Post market Surveillance Under Section 522 of the Federal Food, Drug, and Cosmetic Act,” offering encouragement to manufacturers to have systems that monitor, and report adverse events associated with their products (FDA, 2022b; FDA, 2022c). In addition to this, the FDA increased its enforcement actions against companies that make false or misleading claims about their product’s safety; it remains the line of defense against companies indicating their products cure or treat disease.

Although, FDA investigates adverse event reports and complaints from consumers, healthcare professionals, other regulatory agencies, and industry, the information about the post-market safety of dietary supplements is still limited.

### 1.5 What are the current regulations in Europe?

Like the dietary supplements in the US, the food supplement market has enormously grown in Europe. To date, the European Union legislation does not include a provision to establish a dedicated nutrivigilance system for food supplements (Vo Van Regnault et al., 2021). In Europe, few countries have established their own national surveillance system: Italy (2002), France (2009), Denmark (2013), Portugal (2014), Czech Republic (2015), Slovenia (2016), and Croatia (2020).

Nutrivigilance encounters problems in Europe, where the free movement of goods across borders can allow dietary supplements that are acceptable in one country to enter the market of a country where they have not been evaluated. In other words, where some individual nations may have mechanisms of supplement evaluation and pre-market consumer notification, the overall European Union does not provide such legislation or regulation as a broad umbrella of protection. The collection of EU data and harmonization of nutrivigilance practice is needed from the public health perspective (Vo Van Regnault et al., 2021; de Boer et al., 2022; Wróbel et al., 2023).

## 2 Discussion

Since the reporting of safety concerns and adverse events by consumers is voluntary, manufacturers of dietary supplements stake holders should inform consumers on how to report their safety concerns and adverse events and encourage them to make such reports. On the other hand, this could lead to a voluntary response bias. However, an in-dept analysis could help assess the current situation or risk. A nutrivigilance system, capturing information spontaneously reported from the markets, or evaluation of the cumulative safety data from the manufacturer’s database helps to confirm the safety of products. In a post-marketing surveillance study conducted by Banach et al., nutrivigilance process was used to monitor the reporting rate and nature of the adverse events suspected to be associated with the company’s red yeast rice product (Banach et al., 2021). They found that despite the increase in case reports, the number of reports mentioning serious adverse events due to this product has remained unchanged over the years.

If nutrivigilance is to gain ground as an important means of consumer protection, the movement cannot rely primarily on manufacturers to report adverse effects due to an inherent conflict of interest. For nutrivigilance to succeed, and especially focused on post-market analysis, there must be a fully committed national surveillance system for nutraceuticals and consumers must have awareness of the spontaneous reporting system.

The regulatory authorities, health providers and patients should observe the adverse effects of the nutraceuticals and they need to proactively report the adverse effects related to their consumption. Everyone has a role in the rational and safe use of these in terms of public health. To increase awareness on the topic, pharmacovigilance and adverse effect reaction reporting must be added to the curriculum of health programs. Additionally, academic institutions, policymakers and companies should collaborate to form public health campaigns to increase consumer awareness.
